# Interactions of BDNF Val66met and dietary indices in relation to metabolic markers among patient with type 2 diabetes mellitus: a cross-sectional study

**DOI:** 10.1186/s41043-023-00375-5

**Published:** 2023-04-18

**Authors:** Zeinab Naeini, Faezeh Abaj, Masoumeh Rafiee, Fariba Koohdani

**Affiliations:** 1 Department of Cellular and Molecular Nutrition, School of Nutritional Sciences and Dietetics, Tehran University of Medical Sciences (TUMS), Tehran, Iran; 2 Department of Community Nutrition, School of Nutritional Sciences and Dietetics, Tehran University of Medical Sciences (TUMS), Tehran, Iran; 3 Department of Clinical Nutrition, School of Nutrition and Food Science, Isfahan University of Medical Sciences (IUMS), Isfahan, Iran

**Keywords:** BDNF Val66met, Dietary indices, BMI, Inflammation, Oxidative stress

## Abstract

**Background:**

Gene–diet interaction is related to the progression of diabetes and cardiovascular diseases biomarkers. We aimed to evaluate the interaction between diet quality indices and BDNF Val66Mat (rs6265) on cardiometabolic markers among diabetic patients.

**Methods:**

This cross-sectional study was conducted on 634 patients with type 2 diabetes mellitus, which were randomly recruited from diabetic centers in Tehran. Dietary intakes were estimated by a previously validated semi-quantitative food frequency questionnaire comprising 147 items. All participants were categorized into three categories, based on healthy eating index (HEI), diet quality index (DQI), and phytochemical index (PI) scores. Polymerase chain reaction was used for genotyping the BDNF Val66Met. Interactions were tested using analysis of covariance in adjusted and crude models.

**Results:**

Our result showed that higher DQI, HEI, and PI scores significantly decrease body mass index and waist circumference among individuals with Met/Met, Val/Met, and Val/Val genotypes (*P* interactions < 0.05). Moreover, the highest quartile of the DQI and PI, compared to the lowest, showed lower TG level among Met allele carriers compared to Val/Val homozygotes (*P* interaction = 0.004 and 0.01, respectively) and a faster reduction in IL-18 and TC level was seen among Met/Met, Val/Met who had higher HEI intake than those with Val/Val genotype.

**Conclusions:**

BDNF Val66Met polymorphism may interact with HEI, DQI, and PI. We have revealed that Met allele acts as a protective allele for diabetic patients and may have a beneficial influence on cardio-metabolic factors through regulating dietary intake.

## Background

Type 2 diabetes mellitus (T2DM) as a major multi-factorial chronic health concern is rising rapidly worldwide, and cardiovascular diseases (CVDs) are known as the most common disorders and the main cause of death among patients with T2DM [1]. Conclusive evidence suggests a genetic basis for T2DM and the development of its complications [2]. One of the key target genes which might be involved in cardiometabolic functions via different mechanisms is brain-derived neurotrophic factor (BDNF), which plays a significant role in the regulation of eating behavior and energy expenditure among both animals and human via inhibition of food intake and increasing physical activity [3, 4]. It is thought that Met-allele carriers are related to higher intake of carbohydrate, protein, and total energy and contributes to the modulation of insulin, leptin, ghrelin, and pro-inflammatory cytokines levels [5]. However, some studies found no association [6]. Since eating habits play a central role in the onset and progression of chronic diseases, especially T2DM and CVDs, significant improvement in diabetic dyslipidemia, obesity, and other cardiometabolic risk factors can be achieved via diet management [7]. To determine the nutritional status of individuals, changes in food choices and consumption, and their effect on health, overall diet quality indices have been developed [8]. Healthy eating index (HEI) and diet quality index-international (DQI-I) encourage people to have a diversified, balanced, and healthy diet [9], and phytochemical index (PI), shows the amount of phytochemicals intake in a diet [10].

A few studies have evaluated the association between DQI and HEI with CVDs risk factors among diabetic patients. An inverse relationship between HEI and obesity but no relationship between DQI and body mass index (BMI) has been reported in a recent review among Chinese people [11]. Also, two different Iranian research demonstrated an inverse link between higher HEI scores with a lower risk of obesity and an increased risk of metabolic syndrome [12]. It seems that higher HEI scores which reflect a healthy dietary pattern rich in plant foods and high fiber content play an important role in lowering abdominal obesity, BMI, and waist circumference (WC) [13]. Conversely, a study on elderly Iranian people reported reduced CVDs risk factors among individuals with higher HEI scores [14]. Moreover, a cross-sectional study on diabetic women showed no association between HEI, DQI, and CVDs risk factors [15]. These contradictory results might be related to genetic variations [16]. Since numerous studies highlighted gene-based personalized dietary recommendations for improving health outcomes [17], more gene–diet interactions are needed to focus not only on the association between macro- and micronutrient intake and genetics but also on the impact of dietary patterns on genetics and metabolic health. However, there are limited studies investigating the interactions between BDNF Val66Met and dietary intake on metabolic markers. A research revealed lower prevalence of diabetes and its complication among adults with Val/Met genotype who had low energy and protein intake [18]. A recent interaction study on diabetic patients indicated higher TG and lower HDL-C levels among Val/Met carriers with higher dietary antioxidant capacity [5]. To our knowledge, there is no research investigating the interaction between HEI, DQI, PI, and BDNF polymorphisms on anthropometric and cardiometabolic risk factors. This study aimed to investigate the possible interactions between BDNF Val66Met and dietary indices on metabolic markers among T2DM patients in Iran.

## Methods

### Study design and subjects

This study is part of a larger investigation in which 634 T2DM patients (252 men and 382 women) aged 35–65 years were randomly recruited from diabetes centers such as the Iranian diabetes society and other health centers in Tehran [19]. This group is defined as having either fasting blood sugar (FBS) levels ≥ 126 mg/dl and consuming glucose-lowering medicines. The exclusion criteria were as follows: being under 35 or over 65 years old, insulin-using patients, pregnant, or lactating women.

General information including age, gender, job, smoking and alcohol abuse, lipid-lowering drug consumption, family history of T2DM, and other diseases was collected through interviews. Anthropometric data (body mass index (BMI) and WC) and physical activity (METs) information were taken according to standard protocols [19]. Physical activity was calculated as the metabolic equivalent of task (MET h/day) [20] by a validated and reliable physical activity questionnaire [21]. The study was conducted based on the Declaration of Helsinki and was approved by the ethics committee of Tehran University of Medical Sciences (TUMS) (no. 15060), and the written consent form was obtained from all participants.

### Assessment of dietary intake and indices

The participant’s usual dietary intake during the last year was evaluated through face-to-face interviews with a trained dietitian and using a semiquantitative food frequency questionnaire (FFQ) for 147 food items. This questionnaire was validated by Esmaillzadeh et al. [22]. The subjects were asked to report the frequency of food item consumption in a day, a week, a month, or a year. The amounts listed for each food were converted to grams per day using household measures [19]. Nutritionist III software (version 7.0, N-Squared Computing) was employed to assess energy and nutrient intake.

Three dietary indices were used for evaluating diet quality including the Healthy Eating Index (HEI), assessing dietary intake according to the 2015–2020 dietary guidelines for Americans (DGA) based on a 1000 kcal/day diet. HEI-2015 score ranged from 0 to 100 and consisted of 13 components (vegetables, fruits, beans, seafood or plant-based and total protein foods can receive a score ranging from 0 to 5, whole grains, dairy, fatty acids can receive a score ranging from 0 to 5 and refined grains, saturated fats, sodium, and added sugars are moderated) (higher intakes receive lower scores) [23]. We calculated the score based on responses from the FFQs.

The Diet Quality Index-International (DQI-I) was a second dietary measurement that focuses on four main aspects of a healthy diet (variety, adequacy, moderation, and overall balance). The score for each category is calculated as the sum of the scores for each component in that category. Overall food group variety includes meat/poultry/fish/eggs/dairy/beans/grain/fruit/vegetable (0–15 points), and within-group variety for protein source includes meat/poultry/fish/dairy/beans/eggs (0–5 points), protein sources, vegetable, fruit, grain, fiber, protein, iron, calcium, vitamin C group (0–5 points), moderation foods such as saturated and total fat, cholesterol (CL), sodium and junk foods (0–30 points), macronutrient, and fatty acid (0–10 points) [24]. The total DQI-I score (ranging from 0 to 100 points) is the sum of the scores for the four categories.

Moreover, phytochemical index (PI) is known as the percentage of calorie intake derived from foods rich in phytochemicals including fruits, vegetables, whole grains, nuts, seeds, vegetable juices, soy products, and olive oil. The dietary phytochemical index (DPI) was calculated according to the modified method previously developed by McCarty [25]; [PI = (phytochemical-rich foods g/d/ total food intake g/d) × 100].

### Biochemical assessment and genotyping

An overnight fasting venous blood sample was collected for each subject; the total antioxidant capacity (TAC) [26] of serum was measured by spectrophotometry. It evaluates the overall power of all antioxidants in the body [27]. Serum enzymatic activity of superoxide dismutase (SOD), known as an enzymatic antioxidant [27], was assessed by colorimetric method (Cayman Chemical Company, USA). Interleukin-18 (IL-18), pentrexin-3 (PTX3), and 8-isoprostane F2α (PGF2α) were measured using ELISA method (Shanghai Crystal Day Biotech Co., Ltd). The sensitivity of IL-18 and PTX3 ELISA kit was 28 ng/l and 0.05 ng/ml, respectively. Genomic DNA was isolated from whole blood using salting-out extraction method [28]. Polymerase chain reaction (PCR) was used for genotyping the BDNF Val66Met, followed by 8% polyacrylamide gel electrophoresis. PCR amplification of rs6265 polymorphism was performed by the following primers: forward, 5′-CACTAGCCCAGAGAGAGGAGTG-3′, Reverse, 50-TGAGCCCAGCCGCACACTAAC.

### Statistical analysis

Normal distribution of data was measured using Kolmogorov–Simonov test. Logarithmic transformations were applied to variables with skewed distribution. Participants were divided into two groups: those with Val/Val and Val/Met genotypes versus those of Met/Met homozygotes (Met/Met group). The data were presented as frequency (%) for categorical variables and as mean ± SD for continuous variables. An independent T test was used to compare the quantitative variables between the two groups, and Chi-square test was used to compare the qualitative variables.

The association between diet quality indices and anthropometric or biochemical parameters was evaluated by one-way analysis of variance (ANOVA). The interactions between BDNF Val/Met polymorphism and DEI, DQI, and PI on BMI, WC, TC, HDL-C, LDL-C, LDL/HDL, TG, CRP, PTX3, IL18, TAC, SOD, PGF2α, leptin, and ghrelin were tested using analysis of covariance (ANCOVA) test in two multivariate interaction models, before and after adjustment for potential confounders including age, sexuality, smoking, alcohol consumption, and physical activity. The data were analyzed by IBM SPSS (SPSS Inc., Chicago, IL, USA, version 26), and *P* value < 0.05 was considered statistically significant.

## Results

This cross-sectional study was conducted on 634 T2DM patients with sex distribution of 39.7% and 60.3% in men and women, respectively. The prevalence of the Val66Met genotype among study participants was as follows: The Met allele carrier group had a frequency of 44.2% (Val/Met + Met/Met), and accordingly, the major allele frequency was 55.8% (Val/Val). Anthropometric, general characteristics, nutrient intakes, and comparison of clinical parameters among participants according to BDNF Val66Met genotypes are presented in Tables 1 and 2, respectively. Leptin level was significantly higher among Met allele carriers, while the ghrelin concentration was significantly higher among Val homozygotes (*P* = 0.008 and *P* = 0.02, respectively). Although we found no significant difference regarding other clinical and general parameters across BDNF Val66Met genotypes among the groups, it was seen that Met allele carriers (Met/Met + Val/Met) had nominally significantly higher energy intakes and correspondingly higher intakes of all macronutrients (fat, protein, and carbohydrate) than those with Val/Val genotype.

**Table 1:** Characteristics of patients with type 2 diabetes mellitus

	Val–Met/Met–Met *n* = 280	Val–Val *n* = 354	*P* value
AGE, year	53.69 ± 6.71	54.36 ± 6.30	0.09ª
Weight, kg	77.44 ± 14.42	74.82 ± 13.25	0.10ª
Height, cm	162.19 ± 9.57	160.11 ± 8.89	0.12ª
BMI, kg/m^2^	29.4 ± 4.64	29.18 ± 4.64	0.7ª
Waist circumference, cm	92.52 ± 10.13	91.98 ± 10.8	0.5ª
Physical activity	37.76 ± 6.11	38.03 ± 4.95	0.19ª
Family history	232 (82.9%)	283 (79.9%)	0.3^b^
Heart disease history	104 (37.1%)	138(39%)	
Glucose-lowering medications, no. (%)			
Without medications	20 (7.1%)	23 (6.5%)	0.7^b^
Metformin	20 (7.1%)	104 (29.4%)	
Glibenclamide	14 (5%)	21 (5.9%)	
Metformin + Glibenclamide	132 (47.1%)	181 (51.1%)	
Lipid-lowering medications, no. (%)	162(57.9%)	193 (54.5%)	
Without medications	118 (42.1%)	161 (45.5%)	0.4^b^
Atorvastatin	117 (48.9%)	157 (44.4%)	
Simvastatin	3 (1.1%)	3 (0.8)	
Energy, kcal/day	2333 ± 589.6	2160 ± 687.9	0.1ª
Protein, g/day	91.8 ± 25.1	80.8 ± 26.5	0.9ª
Fat, g/day	100.9 ± 36.1	89.2 ± 32.8	0.4ª
Carbohydrate, g/day	329.5 ± 92.9	317 ± 119.7	0.1ª
Saturated fatty acids, g/day	27.7 ± 8.96	23.35 ± 8.31	0.1ª
Cholesterol, g/day	258.19 ± 118.98	170.03 ± 62.86	0.0ª
Monounsaturated fatty acids, g/day	35.06 ± 14.3	30.95 ± 13.27	0.4ª
*n* − 3 PUFA, g/day	24.7 ± 11.71	22.35 ± 11.17	0.4ª
Fiber, g/day	41 ± 19.09	39.65 ± 18.46	0.9ª

Data are mean ± SD

*P* < 0.05 was considered significant

*BMI* body mass index

^a^Using the independent *t* test

^b^Using the Chi-squared test

**Table 2:** Comparison of clinical and laboratory parameters of participants according to BDNF Val66Met genotypes

	Val–Met/Met–Met *n* = 280	Val–Val *n* = 354	*P* value
CRP (mg/L)	2.06 ± 1.54	2.41 ± 1.46	0.4ª
PTX3 (ng/ml)	2.61 ± 0.51	2.65 ± 0.49	0.6ª
IL-18 (ng/ml)	250.19 ± 29.6	246.55 ± 27.2	0.4ª
TAC (g/dl)	2.55 ± 0.58	2.46 ± 0.57	0.6ª
SOD (U/ml)	0.14 ± 0.04	0.14 ± 0.04	0.7ª
PG2A (ng/ml)	71.23 ± 5.74	73.48 ± 6.67	0.2ª
Leptin (ng/ml)	26.32 ± 16.18	24.52 ± 13.19	0.008
Ghrelin (ng/ml)	2.27 ± 1.14	2.54 ± 1.50	0.02
TG (mg/dl)	173.1 ± 90.98	178.8 ± 93.77	0.8
TCH (mg/dl)	192.21 ± 59.28	196.36 ± 63.45	0.3
LDL-C (mg/dl)	106.27 ± 34.69	107.26 ± 32.74	0.5
HDL-C (mg/dl)	52.21 ± 12.30	53.68 ± 11.78	0.5
LDL/HDL	2.08 ± 0.67	3.06 ± 13.34	0.05

Data are mean ± SD

*CRP* C-reactive protein, *PTX* pentrexin3, *IL-18* Interleukin18, *TAC* total antioxidant capacity, *SOD* superoxide dismutase, *PGF2A* prostaglandin F2-alpha, *TG* triglyceride, *TCH* total cholesterol, *LDL-C* low-density lipoprotein, *HDL-C* high-density lipoprotein. *P* < 0.05 was considered significant

^a^Using the independent *t* test

### Association between cardiometabolic markers and dietary indices

All participants were categorized into three tertiles, based on HEI, DQI, and DPI scores. Analysis for general and biochemical markers between the tertiles is presented in Table 3. Patients in the first quartile of HEI, DQI, and DPI were more likely to be obese. They had higher BMI levels (*P* = 0.01, *P* = 0.02, and *P* = 0.03, respectively). Also, WC level was higher among patients in the first quartiles of HEI and DPI (*P* = 0.01 and *P* = 0.08, respectively). Patients in the last quartile of HEI and DPI had higher levels of LDL-C (*P* = 0.007 and *P* = 0.00, respectively). Moreover, a higher level of HDL-C was seen among the patients in the last quartile of HEI (*P* = 0.01). Regarding HEI, greater adherence reduced the inflammatory markers. There were lower PGF2α (*P* = 0.03) and PTX (*P* = 0.02) levels among patients in the second and third quartile, respectively.

**Table 3:** General and biochemical markers in different quartiles of diet quality indices

Variable	DQI				HEI				PI			
	*Q*1	*Q*2	*Q*3	P*	*Q*1	*Q*2	*Q*3	P*	*Q*1	*Q*2	*Q*3	P*
BMI (kg/m^2^)	29.74 ± 5.13	29.30 ± 4.30	28.39 ± 4.24	0.027	30.02 ± 4.72	28.97 ± 4.72	28.77 ± 4.23	0.01	29.87 ± 5.27	29.23 ± 4.4	28.57 ± 4.08	0.03
WC (cm)	92.39 ± 11.18	92.42 ± 9.78	91.51 ± 10.71	0.6	93.85 ± 9.98	91.82 ± 11.19	90.60 ± 9.61	0.01	93.58 ± 10.59	91.67 ± 10.04	91.46 ± 11.14	0.08
CRP (mg/l)	1.89 ± 1.32	2.41 ± 1.58	2.59 ± 1.5	0.1	2.16 ± 1.58	2.21 ± 1.39	2.52 ± 1.59	0.5	2.25 ± 1.33	2.34 ± 1.65	2.16 ± 1.40	0.8
PTX3 (ng/ml)	2.6 ± 0.39	2.71 ± 0.48	2.50 ± 0.68	0.1	2.66 ± 0.38	2.72 ± 0.47	2.43 ± 0.63	0.02	2.61 ± 0.46	2.70 ± 0.48	2.53 ± 0.56	0.2
IL-18 (pg/ml)	253.22 ± 28.35	245.98 ± 27.93	244.01 ± 28.29	0.3	252.5 ± 27.18	246.1 ± 27.95	245.6 ± 30.04	0.4	246.53 ± 25.67	248.61 ± 29.51	248.52 ± 28.81	0.9
TAC (g/dl)	2.52 ± 0.59	2.48 ± 0.56	2.51 ± 0.59	0.9	2.55 ± 0.51	2.50 ± 0.63	2.44 ± 0.56	0.7	2.45 ± 0.52	2.52 ± 0.57	2.52 ± 0.64	0.8
PGF2A (pg/ml)	72.45 ± 5.5	72.31 ± 7.25	73.36 ± 5.54	0.7	74.34 ± 6.49	71.37 ± 6.52	70.26 ± 5.41	0.03	71.97 ± 6.35	72.88 ± 6.21	72.52 ± 6.58	0.7
SOD (U/ml)	0.15 ± 0.047	0.14 ± 0.04	0.14 ± 0.04	0.5	0.14 ± 0.05	0.15 ± 0.04	0.13 ± 0.03	0.4	0.14 ± 0.04	0.14 ± 0.04	0.14 ± 0.04	0.9
TG (mg/dl)	182.73 ± 97.58	171.86 ± 90.66	173.79 ± 86.68	0.3	173 ± 93.80	180.48 ± 94.43	173.28 ± 87.13	0.6	183.29 ± 91.99	173.16 ± 92.9	173.17 ± 92.57	0.4
TCH (mg/dl)	200.42 ± 66.09	195.10 ± 59.71	188.79 ± 56.48	0.05	194 ± 66.48	192.74 ± 59.59	198.67 ± 58.24	0.6	198.6 ± 68.31	192.72 ± 60.76	188.59 ± 53.43	0.04
LDL-c (mg/dl)	107.91 ± 31.88	104.90 ± 34.43	108.76 ± 34.90	0.4	111.09 ± 30.7	108.69 ± 32.88	101.62 ± 37.75	0.007	112.29 ± 32.12	109.43 ± 33.68	102.12 ± 34.46	0.00
HDL-c (mg/dl)	53.46 ± 12.17	52.44 ± 11.79	53.45 ± 12.29	0.5	51.16 ± 12.43	53.75 ± 11.57	54.39 ± 12.04	0.01	53.32 ± 12.34	52.99 ± 12.26	52.72 ± 11.18	0.8
LDL/HDL	2.08 ± 0.66	2.69 ± 10.50	3.50 ± 16.08	0.4	2.85 ± 11.76	2.07 ± 0.64	3.36 ± 15.23	0.4	2.11 ± 0.63	2.57 ± 10.08	3.43 ± 15.17	0.4
Leptin (ng/ml)	24.94 ± 12.46	26.55 ± 15.59	23.61 ± 14.89	0.4	23.57 ± 12.65	24.42 ± 16.17	28.18 ± 13.07	0.1	24.14 ± 13.96	26.05 ± 15.31	24.80 ± 13.46	0.7
Ghrelin (ng/ml)	2.31 ± 1.32	2.33 ± 1.24	2.75 ± 1.63	0.1	2.35 ± 1.41	2.52 ± 1.40	2.41 ± 1.35	0.7	2.56 ± 1.66	2.31 ± 1.22	2.55 ± 1.38	0.4

Data are mean ± SD

*P* < 0.05 was considered significant

*CRP* C-reactive protein, *PTX* Pentrexin3, *IL-18* Interleukin18, *TAC* total antioxidant capacity, *SOD* superoxide dismutase, *PGF2A* prostaglandin F2-alpha, *TG* triglyceride, *TCH* total cholesterol, *LDL-C* low-density lipoprotein, *HDL-C* high-density lipoprotein

P* the one-way analysis of variance (ANOVA) was used to determine whether there are any statistically significant differences between the parameters and dietary indices among different quartiles

### Interaction between dietary indices with BDNF Val66Met variants on cardiometabolic markers

**Fig. 1:**
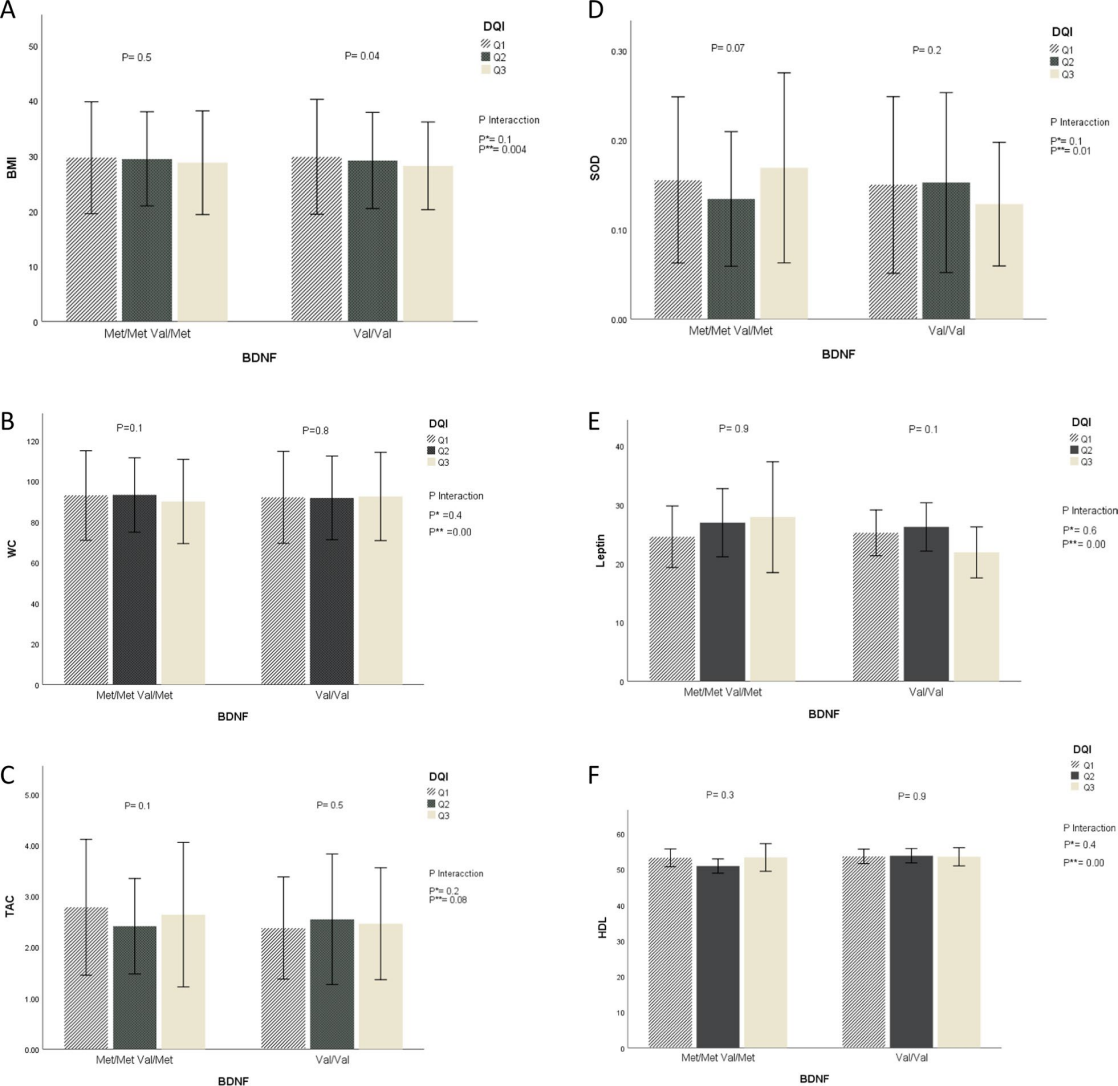
The interaction between BDNF Val66Met genotypes and quartiles of diet quality index (DQI) on: **A** body mass index (BMI), **B** waist circumference (WC), **C** total antioxidant capacity (TAC), **D** superoxide dismutase (SOD), **E** leptin mean, **F** high-density lipoprotein (HDL) and standard error. *P* values for the interaction obtained in two models using ANCOVA. P*: unadjusted; P**: adjusted for age, sexuality, smoking, alcohol consumption, and physical activity

**Fig. 2:**
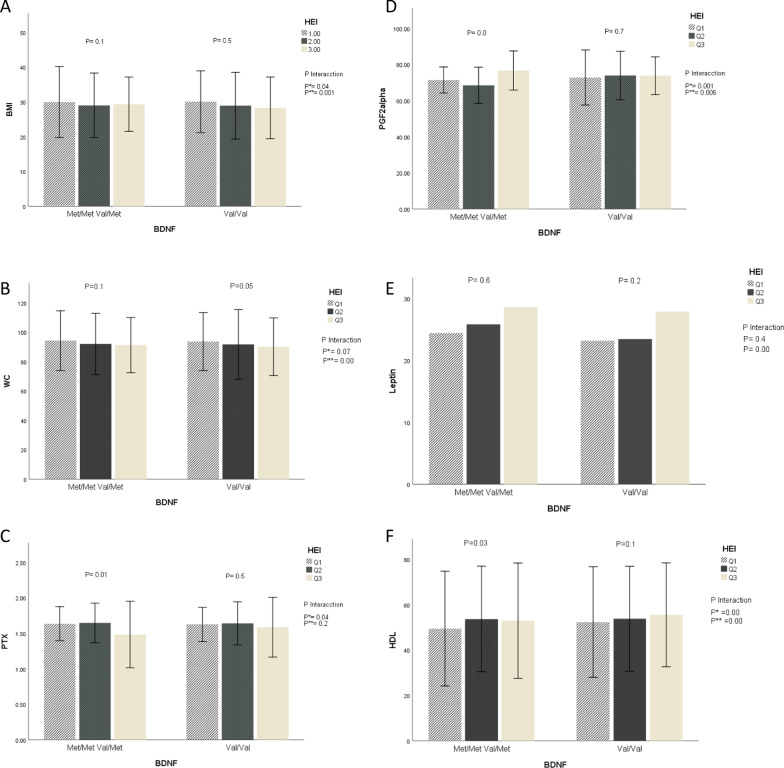
The interaction between BDNF Val66Met genotypes and quartiles of healthy eating diet (HEI) on: **A** body mass index (BMI), **B** waist circumference (WC), **C** pentraxins (PTX), **D** prostaglandin F2 Alpha (*PGF2*) **E** leptin mean, **F** high-density lipoprotein (HDL) mean and standard error. *P* values for the interaction obtained in two models using ANCOVA. P*: unadjusted; P**: adjusted for age, sexuality, smoking, alcohol consumption and physical activity

**Fig. 3:**
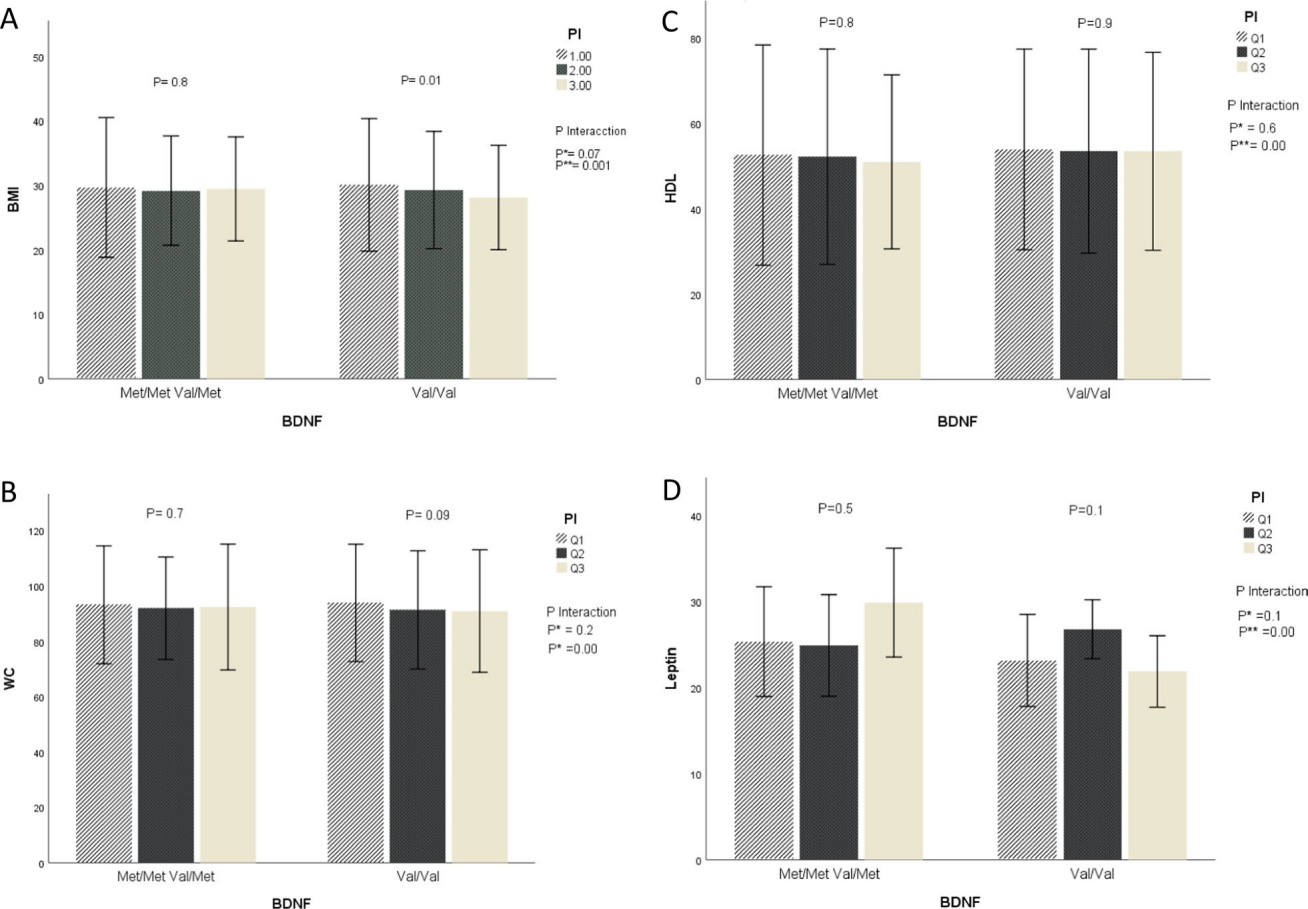
The interaction between B. DNF Val66Met genotypes and quartiles of dietary phytochemical index (PI) on: **A** body mass index (BMI), **B** waist circumference (WC) **C** leptin mean, **D** high-density lipoprotein (HDL) mean and standard error. *P* values for the interaction obtained in two models using ANCOVA. P*: unadjusted; P**: adjusted for age, sexuality, smoking, alcohol consumption, and physical activity

Significant interactions between BDNF Val66Met polymorphism and DQI, HEI, and PI scores on cardiometabolic markers are shown in Figs. 1, 2, and 3. Significant interactions were observed between HEI and Val66Met polymorphism for BMI and WC (*P* = 0.001 and *P* = 0.0, respectively). Our results revealed that higher scores for all diet-quality indices are significantly associated with lower BMI and WC values among all participants. It remained consistently significant even after adjusting for various potential confounders including age, weight, height, and physical activity. Also, interactions between DQI and DPI and Val66Met polymorphism for BMI and WC were significant after adjustment.

The highest quartile of DQI was associated with elevated levels of SOD among individuals with Val/Met and Met/Met (*P* interaction = 0.01).

## Discussion

This cross-sectional study aimed to assess the interactions between BDNF Val66Met polymorphism and HEI, DQI, and DPI on anthropometric and metabolic markers among diabetic patients. We found patients with lower scores of HEI, DQI, and DPI were more likely to be obese. There was a significant decreasing trend in the odds of BMI and WC across increasing quartiles of all measured dietary indices. Also, WC level was significantly lower among diabetic patients with higher scores for HEI and DPI. It is noteworthy that patients in the highest group of DQI and DPI had lower total fat intake including saturated fats and dietary cholesterol and lower TC level compared with those in the lower quartiles. In addition, higher protein intake was observed among the highest quartile of all the indices compared to the lower groups. A few studies which have investigated the association between dietary indices and anthropometric or cardiometabolic risk factors, reported controversial results. The inverse association between diet quality indices and obesity was approved by some previous studies [29, 30]. Quatromoni et al. found an inverse relationship between higher adherence to DQI and lower weight gain in an 8-year cohort study [31]. It has been demonstrated that unhealthy dietary patterns, rich in saturated fatty acids (SFAs), sugar, and red or processed meat which have low scores for HEI promote fat accumulation in different adipose tissues, particularly abdominal fat which is strongly related to the risk of metabolic disorders and atherogenesis [32]. However, the exact mechanism of diet quality scores on BMI values is not fully understood [33].

It should be considered that monitoring the overall eating patterns which reflect the intake of all food groups is more effective for finding about diet quality than investigating the effects of particular micro- or macronutrients [34]. Surprisingly, we observed a significant decrease in BMI and WC among those who had higher energy intake. However, higher scores for HEI were directly associated with higher energy and fat intake and showed a healthier dietary pattern among patients including lower intake of saturated fats and higher consumption of protein and healthier oils rich in polyunsaturated fatty acids (PUFAs) when compared to those with lower scores who consumed less total energy but more saturated fatty acids and cholesterol. Several studies have shown higher HEI scores are directly linked to healthier lifestyles and eating behaviors [35, 36]. As previous studies suggested, saturated fatty acid intake has been known as important nutrient responsible for weight gain in modern societies and is inversely associated with WC [37]. It has been suggested that the saturation level of fats in the diet contributes to the rate of fat storage as well as the amount of fat intake [10, 38]. A study that examined the effect of fatty acid composition of the diet in mice, revealed a diet rich in PUFAs prevents myocellular lipid accumulation. It showed that the highest increase in BMI was associated with a diet rich in palmitic acid (C16:0) [10]. This finding was in line with other reports indicating that there is a preference for oxidizing PUFAs due to oxidizing more rapidly than saturated fatty acids, while saturated fat seems to be stored in muscle rather than oxidizing for energy [39, 40]. It has been shown that TG composition in adipose tissue reflects habitual dietary fat intake over a short and long time [41, 42]. Our result would be another approval for this relationship. As expected, higher PUFAs and lower SFAs intake among diabetic patients in the highest quartile of HEI were also associated with a decrease in levels of LDL-C and inflammatory markers (PTX, and PGF2α) and an increase in HDL-C. Although some research among Iranians reported no significant association between HEI/AHEI and lipid profile, CRP level, and other CVDs risk factors [43, 44], several reported little association between DQI or HEI and inflammatory biomarkers [45] or showed this relationship only among men, not women [35].

To our knowledge, the association between diet quality indices and BMI or metabolic markers has been widely investigated. However, a few recent studies have highlighted gene-nutrition interaction on health outcomes [5, 42]. Thus, the current study aimed to investigate the possible interplay between dietary indices (HEI, DQI, and DPI) and BDNF Val66Met polymorphism in relation to cardiometabolic markers among patients with T2DM.

We present novel findings regarding the interaction between HEI, DQI, DPI, and BDNF Val66Met polymorphism on BMI and WC levels. Moreover, we found significant interactions between DQI and Val66Met polymorphism on SOD, HDL, and leptin, between HEI and the Val66Met polymorphism on PGF2α, HDL, and leptin levels, and between DPI and this polymorphism on HDL levels. We observed BDNF variants affected daily dietary intake such as total energy, carbohydrates, fiber, protein, and fat in diabetic patients. Generally, Met allele carrier group had significantly higher nutritional intake compared to subjects with Val/Val genotype. It might be related to their leptin level, which basically was higher among Met allele carriers. They were also more likely to increase their leptin level in higher scores of HEI and DQI. Further major finding is related to a significant decrease in cardiometabolic risk factors among diabetic patients with higher adherence to HEI and DQI. Met allele carriers were more likely to have experienced a faster reduction in IL-18 and TC levels by having higher HEI intake compared to those with Val/Val genotype. Interestingly, Met allele carriers who consumed diets higher in DQI had a significant decrease in TG level, while it showed an increase for those carrying Val/Val. Several studies have reported that the relationship between T2DM and BDNF Val66Met depends on diet [18, 46]. They found a positive relationship between BDNF and obesity or type 2 diabetes development in humans due to its role in eating behavior and energy homeostasis. Xian-Yong Ma et al. reported a significant interaction between BDNF Val66Met and PUFAs intake on elevated risk of obesity [47].

Abaj et al. showed a significant interaction between dietary insulin index and dietary insulin load scores and BDNF Val66Met on BMI level [48]. Consistent with our result, a study showed that the risk of T2DM was lower among participants with Val/Met genotype even with a diet rich in carbohydrates, while Val/Val participants with lower energy intake were more likely to develop diabetes [18]. Previous studies that first demonstrated in rats showed that BDNF modulates metabolic function by affecting appetite and controlling food intake, energy metabolism, and insulin sensitivity [49, 50]. Moreover, BDNF contributes to the regulation of energy metabolism through peripheral neurons which are involved in the maintenance of energy balance [51]. It has been shown previously that hyperphagia, obesity, and metabolic imbalances are related to BDNF deletion in both knockout mice and humans [52, 53]. So, it is not surprising that there is a lower circulating BDNF among diabetic patients and individuals with obesity or metabolic disorder compared with healthy people [54, 55]. Nakagawa et al. revealed that heterozygous BDNF knockout mice are hyperphagic and obese and weight loss due to appetite suppression has occurred in BDNF-infused diabetic mice [56]. Furthermore, Met allele carriers have been shown to have lower plasma BDNF levels compared to Val/Val homozygotes [57, 58]. On the other hand, BDNF expression can regulate by nutritional intake. A recent study on rodents reported higher BDNF expression after a high-fat diet [59]. Gyorkos et al. found that a carbohydrate-restricted diet increases BDNF levels in Met allele carriers [53]. Moreover, a recent study showed high glycemic index foods have a negative effect on cardiometabolic factors among Val/Val participants possibly due to lowering BDNF levels [48]. Studies found a protective effect among Met allele carriers even in high-energy and high-carbohydrate diets [18, 48]. The present study revealed the positive effect of high HEI and DQI on lipid profile especially among Met allele carriers when compared to Val/Val. Although the intake of SFAs and CL has been decreased and PUFAs intake has been increased among both genotypes in higher quartiles for HEI, we revealed that increased HEI did not influence on negative consequences of Val/Val genotype in terms of TG and CL levels. Similar to what has been seen among Val/Val carriers with higher DQI and DPI intake. Despite increased fiber intake and decreased cholesterol consumption among both genotypes, IL-18 and TG levels decreased only among Met carrier group. Moreover, we have seen that increased physical activity and fiber intake in higher DPI scores could decrease TAC, SOD, and PGF2α levels in Met/Met and Val/Met, while these factors have been increased among individuals with Val/Val genotype even after having less fat intake. We found an especially interesting protective impact for Met allele among diabetic patients. It is suggested that diet quality indices may regulate the association between BDNF Val66Met and cardiometabolic factors by making changes in BDNF expression.

The main limitation of this research is that we could not measure the blood sugar and serum BDNF levels of participants due to time and budget limitations. So, we missed potentially relevant interactions. Besides, although we controlled for several potential confounders, the effects of remaining confounders cannot be ignored. Thus, further investigations in different populations are needed to confirm the findings.

## Conclusions

The present study revealed a protective effect of Met allele against cardiometabolic markers through modulating dietary patterns among individuals with T2DM. Participants with Met/Met and Val/Met genotypes who had higher DQI and DPI intake have experienced a significant decrease in TG level, while it has increased in homozygous patients for Val allele. Moreover, a major decrease in BMI levels among all patients with higher adherence to HEI, DQI, and DPI was seen. However, it has been shown that consuming higher PUFAs is much more effective for lowering the risk of obesity rather than lower energy and fat intake, especially among those with higher HEI.

## Data Availability

The data are not publicly available due to containing private information of participants. Data are available from the authors upon reasonable request. Correspondence and requests for materials should be addressed to F.K. and M.R.

## Abbreviations

CVDs: Coronary vascular diseases
HEI: Healthy eating index
DQI: Diet quality index
PI: Phytochemical index
PCR: Polymerase chain reaction
BMI: Body mass index
WC: Waist circumference
SNPs: Single-nucleotide polymorphisms
BDNF: Brain-derived neurotrophic factor
DQI-I: Diet quality index-international
PI: Phytochemical index
DPI: Dietary phytochemical index
FBS: Fasting blood sugar
TAC: Total antioxidant capacity
SOD: Superoxide dismutase
IL-18: Interleukin-18
PTX3: Pentrexin-3
PGF2α: 8-Isoprostane F2α
PUFAs: Polyunsaturated fatty acids
SFAs: Saturated fatty acids
CL: Cholesterol
